# GSH-doped GQDs using citric acid rich-lime oil extract for highly selective and sensitive determination and discrimination of Fe^3+^ and Fe^2+^ in the presence of H_2_O_2_ by a fluorescence “turn-off” sensor

**DOI:** 10.1039/c7ra13432k

**Published:** 2018-03-14

**Authors:** Khanitta Saenwong, Prawit Nuengmatcha, Phitchan Sricharoen, Nunticha Limchoowong, Saksit Chanthai

**Affiliations:** Materials Chemistry Research Center, Department of Chemistry, Center of Excellence for Innovation in Chemistry, Faculty of Science, Khon Kaen University Khon Kaen 40002 Thailand sakcha2@kku.ac.th; Nanomaterials Chemistry Research Unit, Department of Chemistry, Faculty of Science and Technology, Nakhon Si Thammarat Rajabhat University Nakhon Si Thammarat 80280 Thailand

## Abstract

Synthesis and characterization of graphene quantum dots (GQDs) simultaneously doped with 1% glutathione (GSH-GQDs) by pyrolysis using citric acid rich-lime oil extract as a starting material. The excitation wavelength (*λ*_max_ = 337 nm) of the obtained GSH-GQD solution is blue shifted from that of bare GQDs (*λ*_max_ = 345 nm), with the same emission wavelength (*λ*_max_ = 430 nm) indicating differences in the desired N and S matrices decorating the carbon based nanoparticles, without any background effect of both ionic strength and masking agent. For highly Fe^3+^-sensitive detection under optimum conditions, acetate buffer at pH 4.0 in the presence of 50 μM H_2_O_2_, the linearity range was 1.0–150 μM (*R*^2^ = 0.9984), giving its calibration curve: *y* = 34.934*x* + 169.61. The LOD and LOQ were found to be 0.10 and 0.34 μM, respectively. The method’s precisions expressed in terms of RSDs for repeatability (*n* = 3 × 3 for intra-day analysis) were 2.03 and 3.17% and for reproducibility (*n* = 5 × 3 for inter-day analysis) were 3.11 and 4.55% for Fe^2+^ and Fe^3+^, respectively. The recoveries of the method expressed as the mean percentage (*n* = 3) were found in the ranges of 100.1–104.1 and 98.08–102.7% for Fe^2+^ and Fe^3+^, respectively. The proposed method was then implemented satisfactorily for trace determination of iron speciation in drinking water.

## Abstract 2

Synthesis and characterization of graphene quantum dots (GQDs) simultaneously doped with 1% glutathione (GSH-GQDs) by pyrolysis using citric acid rich-lime oil extract as a starting material.

## Graphical abstract

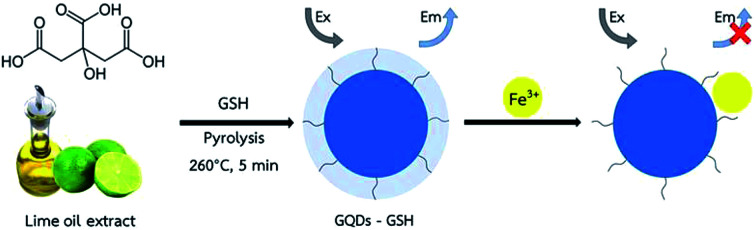

## 1. Introduction

Iron (Fe) is a metal of biological, clinical, environmental, and industrial importance. It is one of the most essential trace elements in living biosystems and plays indispensable and versatile roles in many physiological and pathological processes, including enzyme catalysis, oxygen transport, cellular metabolism, electron transfer, and DNA and RNA synthesis.^1–5^ Speciation of iron, an occurrence of the element in two oxidation states (ii and iii), is mostly found in nature. Different biological activities of Fe^2+^and Fe^3+^ are well known. Fe^2+^ is favored for absorption by biological cells. To treat clinical symptoms of iron deficiency some medicaments containing Fe^2+^ can be administrated to eliminate complications. The low stability of Fe^2+^ caused by its easy oxidation to Fe^3+^ by oxygen in the air can result in a decrease in the real Fe^2+^ concentration in pharmaceuticals.^6^ However, both a deficiency and an excess accumulation of iron in the human body can induce serious disorders such as anaemia, intelligence decline, arthritis, heart failure, diabetes and cancer.^7–9^ Thus, the determination of iron speciation is of fundamental importance for the early identification and diagnosis of these diseases. In addition, the measurement of iron concentration in water samples is also crucial for environmental safety.^10^

Currently, several analytical techniques for determination of Fe^2+^ and Fe^3+^ ions have been applied including solid phase extraction,^11^ fiber-optic chemosensor,^12^ voltammetric methods,^13^ high performance liquid chromatography,^14^ capillary electrophoresis,^15^ inductively coupled plasma mass spectrometry,^16^ flow injection analysis^17^ and chemiluminescence.^18^ Although these techniques are highly sensitive and selective, they require tedious sample preparation and preconcentration procedures, expensive instruments, and professional personnel.

In recent years, several fluorescence sensors have been widely investigated for the selective detection of iron speciation in biological systems because of their ability to provide a simple, sensitive, and selective method for monitoring without the need for any pretreatment of the sample; these techniques also have the advantages of spatial and temporal resolution.^19–21^ Therefore, the development of a novel fluorescence probe with low cytotoxicity, excellent biocompatibility, and high water solubility has become increasingly important and urgent. Recently, graphene quantum dots (GQDs) have ignited increasing research interest as an exciting class of carbon nanomaterial, and have emerged as a potential new platform in designing and tuning fluorescence (FL) biosensing and imaging.^22–35^ GQDs were discovered very recently as a class of zero-dimensional graphitic nanomaterials with lateral dimensions less than 100 nm in single layers, double layers and few layers (3 to <10).^36–39^ Moreover, similar to graphene nanosheets, GQDs have excellent characteristics such as large surface area, large diameter, fine surface grafting *via* the π–π conjugated network or surface groups and other special physical properties.^40,41^ Furthermore, the carboxyl and hydroxyl groups at their edge enable them to display the excellent properties of high biocompatibility, low toxicity, chemical inertness and good water-solubility for successive functionalization with various organic, inorganic, polymeric or biological species.^42–46^ On the basis of these unique properties, various GQD-based fluorescence probes for the detection of metal ions (Fe^3+^, Cu^2+^, or Pb^2+^),^35–37^ small organic molecules (2,4,6-trinitrotoluene or *para*-nitrophenol)^47,48^ and biomaterials (pyrocatechol, human immunoglobulin G, or protein kinase)^49–51^ have been explored. The major disadvantage of GQD-based fluorescence probes is that the sensitivity and selectivity are limited due to the non-specificity of GQDs.^52–54^ Doping GQDs with heteroatoms can effectively modulate their band gap and electronic density, enhancing the chemical activity of GQDs for practical applications, which has been proven through theoretical calculations and detailed experiments.^55–59^ For example, nitrogen-doped GQDs (N-GQDs) showed efficient electrocatalytic activity for the oxygen reduction reaction^46^ and cellular and deep-tissue imaging.^60,61^ Boron-doped GQDs (B-GQDs) gave rise to rich fluorescence owing to their peculiar interaction with the surrounding media.^62^ Sulfur-doped graphene quantum dots (S-GQDs) showed a stable blue-green fluorescence, drastically improved electronic properties, and increased surface chemical reactivity compared with undoped GQDs.^63^

Towards this target, a lot of synthesis methods for various functionalized and non-functionalized GQDs have been established in recent years. Some toxic small organic molecules have been employed to fabricate GQDs using multistep oxidative condensation reactions in organic solutions. In most cases, however, these organic synthetic methods suffer from some disadvantages, such as harsh reaction conditions, high prices, tedious processes, and the use of toxic starting materials.^64–68^ In this regard, searching for precursors from regular food may provide green routes that could overcome the above mentioned drawbacks. Some natural foods have been consumed by human beings for centuries, and are still very common in daily life. If natural food could be used as a non-toxic starting material for the synthesis of GQDs, such eco-friendly synthesis would be valuable. Recently there have been a few reports on fabricating fluorescent QDs from natural foods such as rice husk,^69^ honey,^70^ glucose,^71^ milk,^72^ orange juice^73^ and coffee^74^ due to their low cost, easy availability, and nearly unlimited sources.

These results inspired us to further produce fluorescent GQDs from natural foods. In this work, a selective fluorescence sensor for speciation of iron was developed based on graphene quantum dots, functionalized with glutathione (GSH-GQDs) prepared from lime oil extract. The lime oil extract was pyrolyzed in the presence of glutathione (GSH) to prepare N and S co-doped graphene quantum dots (GSH-GQDs). In the presence of ferric ion (Fe^3+^), the fluorescence intensity of the GSH-GQDs decreased linearly with increasing Fe^3+^ concentration due to quenching. The detection of Fe^2+^ is similar to the procedure mentioned for the determination of Fe^3+^ with the difference being the addition of H_2_O_2_ to the sample as an oxidation agent. This is intended to be used as a fluorescence sensor for the determination of Fe^2+^ and Fe^3+^ ions. In addition, the optimum conditions including Fe^2+^, Fe^3+^ and H_2_O_2_ concentration and the pH of the solution were investigated.

## 2. Experimental

### 2.1 Chemicals

All chemicals are of analytical grade. Citric acid, sodium acetate, sodium chloride, iron(ii) sulfide, iron(iii) chloride hexahydrate and paraffin oil were purchased from QRec™ (New Zealand). Sodium hydroxide, silver chloride, magnesium chloride, manganese(ii) sulfate monohydrate, nickel(ii) chloride and aluminium chloride were purchased from Carlo Erba (Italy). l-Glutathione (reduced form), mercury nitrate, cobalt nitrate hexahydrate, lead nitrate, zinc nitrate hexahydrate, cadmium nitrate tetrahydrate, copper nitrate trihydrate, and ethylenediaminetetraacetic acid (EDTA) were purchased from Sigma-Aldrich (Germany). Acetic acid was purchased from Merck (Germany). Hydrogen peroxide was purchased from Ajax Finechem (Australia). A Millipore water purification system (Molsheim, France) was used to obtain deionized water with a resistivity of 18.2 MΩ cm.

### 2.2 Instruments

Spectrofluorometer Model RF-5301PC (Shimadzu, Japan) was mainly used. UV-visible spectrophotometer Model Agilent 8453 was from Agilent (Germany). pH meter UB-10 UltraBasic (Denver, USA) was used for the solution pH buffering system. Attenuated total reflectance-Fourier transform infrared (ATR-FTIR) spectroscopic measurement was performed on a TENSOR 27 system Fourier transform infrared spectrometer (Bruker, Germany). EDX spectra were taken by a HITACHI S-3000N scanning electron microscope (SEM, Hitachi Co. Ltd., Japan).

### 2.3 Preparation and characterization of graphene quantum dots (GQDs) from lime oil extract

GQDs from citrus oil extract were prepared by pyrolysis. In this typical procedure, 130 mL of lime juice was evaporated to obtain lime oil extract. Then 2.0 g of the oil extract was added into a 5 mL beaker. The beaker was heated to 260 °C using a paraffin oil bath for about 5 min. The liquid was then transferred into a beaker containing 100 mL of 0.25 mol L^−1^ NaOH with continuous stirring for 30 min. The obtained sample solution was neutralized to pH 7.0 with NaOH, and the GQD stock solution was stored at 4 °C before use.

### 2.4 Preparation of graphene quantum dots functionalized with glutathione (GSH-GQDs)

GSH-GQDs were also prepared by pyrolysis. Briefly, 2.0 g of lime oil extract and 1% (w/w) glutathione were added into a 5 mL beaker. The beaker was heated to 260 °C using a paraffin oil bath for about 5 min. The lime oil extract and glutathione mixture was slowly liquated and turned a brown colour. The liquid was transferred into a beaker containing 100 mL of 0.25 mol L^−1^ NaOH with continuous stirring for 30 min. The GSH-GQD stock solution was stored at 4 °C before use.

### 2.5 Fluorescence measurement

The fluorescence measurement of GSH-GQDs was performed in acetate buffer solution at pH 4. For the determination of Fe^3+^, 500 μL of GSH-GQD solution and 1 mL of 1.0 M acetate buffer at pH 4 were mixed in a 10 mL volumetric flask. Then, various concentrations of Fe^3+^ were added to an aliquot of the GSH-GQD solution (10 mL final volume) at room temperature. The Fe^3+^ species that quenched the fluorescence intensity of each GSH-GQD solution were recorded immediately at 430 nm when excited at 345 nm. Then, the spectral measurements were used to plot the quenching calibration curve for Fe^3+^. The detection of Fe^2+^ species was also done by a similar procedure to that mentioned for the determination of Fe^3+^ with the difference being the addition of H_2_O_2_ as an oxidizing agent to the sample solution.

### 2.6 Optimization of the proposed fluorescence sensor

To obtain the optimized conditions of the proposed fluorescence sensor, the main experimental parameters affecting the fluorescence intensity of the GSH-GQDs were investigated in detail as follows.

#### 2.6.1 Effect of pH

The effect of the solution pH towards the fluorescence quenching of GSH-GQDs by Fe^3+^ was studied. The experiment was carried out by adjusting 1.0 M buffer solution (pH 2–10) containing 500 μL of GSH-GQD solution and 25 μM of Fe^3+^(or Fe^2+^) to the desired pH solution.

#### 2.6.2 Effect of H_2_O_2_ concentration

The effect of H_2_O_2_ concentration was tested by an evaluation of the fluorescence intensity of the GSH-GQDs. To a 10 mL volumetric flask containing 500 μL of the GSH-GQD solution, various amounts of H_2_O_2_ and Fe^2+^ were added and the solution was adjusted to 10 mL prior to fluorescence measurement in order to achieve solutions with final concentrations of 5, 10, 25, 50, 75 and 100 μM H_2_O_2_ and 100 μM of Fe^2+^. According to the results, the concentration of H_2_O_2_ required for the complete oxidation of Fe^2+^ did not noticeably change the fluorescence intensity of the GSH-GQDs.

#### 2.6.3 Effects of ionic strength and masking agent

Various concentrations of 0.05, 0.10, 0.25, 0.20 and 0.25 M NaCl were added into 100 mg L^−1^ GSH-GQD solution and adjusted to 10 mL in a volumetric flask prior to fluorescence measurement. In the same manner, 0.01, 0.02, 0.03, 0.04 and 0.05 M EDTA was added into the GSH-GQD solution and fluorescence spectra were recorded. The results were compared and discussed.

#### 2.6.4 Selectivity of the proposed fluorescence sensor

To evaluate the selectivity of the proposed fluorescence sensor, the following procedure was carried out. An individual stock solution of various metal ions (10 mM) was prepared by dissolution of a metal salt in deionized water. To a 10 mL volumetric flask containing 500 μL of the GSH-GQD solution was added 0.1 mL of 10 mM of the metal ion (final concentration of 100 μM) and the solution was adjusted to 10 mL with deionized water prior to fluorescence measurement.

#### 2.6.5 Method validation of the proposed fluorescence sensor

The proposed fluorescence sensor was validated according to the following analytical features of merit: linearity, limit of detection (LOD), limit of quantitation (LOQ), precision (% RSD), and accuracy (% recovery).

## 3. Results and discussion

### 3.1 Characterization of the as-synthesized GQDs and GSH-GQDs

**Fig. 1:**
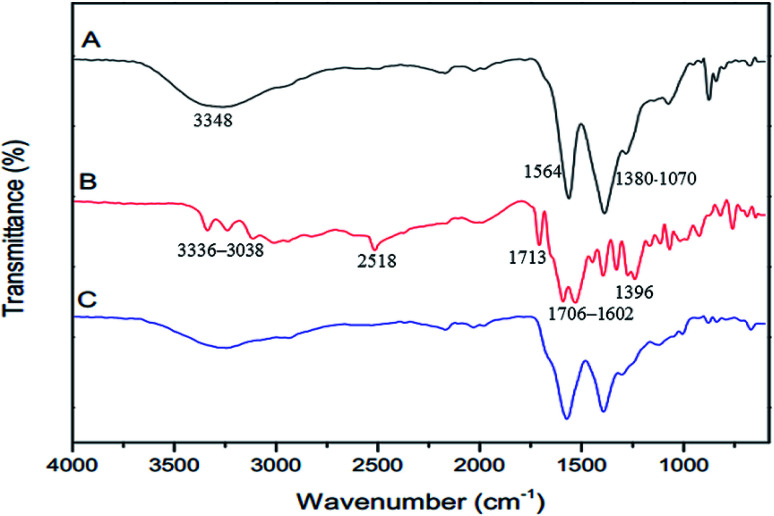
FTIR spectra of (A) GQDs, (B) GSH and (C) GSH-GQDs.

The FTIR spectra of GQDs, GSH, and GSH-GQDs are shown in Fig. 1. The as-prepared GQDs (Fig. 1A) show peaks at 1564 cm^−1^, which indicates the presence of the C=C stretching mode of polycyclic aromatic hydrocarbons.^75^ A broad peak at 3348 cm^−1^ is the characteristic peak of the hydroxyl group (–OH) from water molecules and carboxylic groups. The 1380–1070 cm^−1^ are attributed to the C–O in COH/COC (epoxy) groups.^76^ These results confirm the GQDs were successfully synthesized by lime oil extract pyrolysis. The FTIR spectrum of GSH is shown in Fig. 1B. Characteristic GSH broad absorption bands around 1706–1602 cm^−1^ (symmetric COO^−^), 1396 cm^−1^ (asymmetric COO^−^) and 1713 cm^−1^ (C=O) are observed indicating a –COOH group is present. Peaks around 3336–3038 cm^−1^ indicate symmetric N–H stretching and 2518 cm^−1^ indicate –SH (S–H) groups,^77^ respectively. In the FTIR spectra of GQDs functionalized with GSH (Fig. 1C), the disappearance of the S–H group vibration at 2518 cm^−1^ (S–H) is clear and is likely a consequence of a covalent bond established between the GQDs and GSH. These spectra confirmed the presence of GSH doped in the GQDs.

**Fig. 2:**
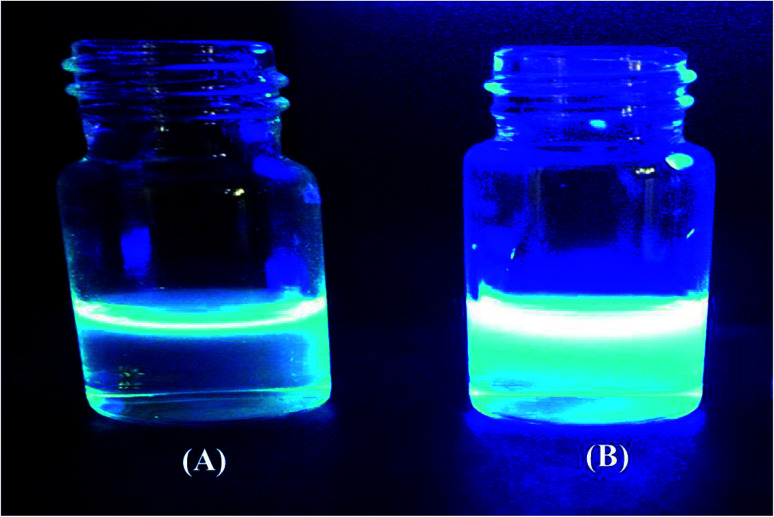
The blue emission of (A) GQDs and (B) GSH-GQDs under UV light.

**Fig. 3:**
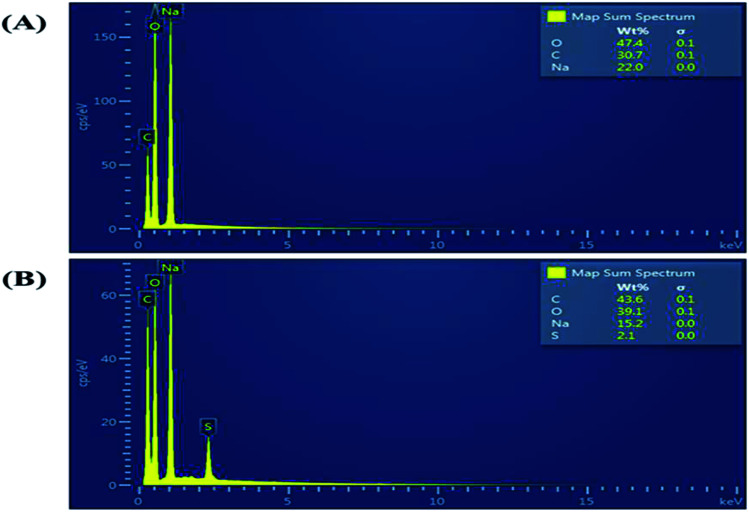
EDX spectra of (A) GQDs and (B) GSH-GQDs.

Fig. 2 shows a photograph of the solution of GQDs Fig. 2(A) and GSH-GQDs Fig. 2(B) taken under UV light. The GSH-GQD solution shows brighter blue emission than the GQDs under UV light. It was also confirmed by EDX spectra (Fig. 3A and B) that the GSH doped GQDs, compared with bare GQDs, were obtained by the appearance of S at 2.31 keV (Fig. 3B) which was commonly found in association with C (0.277 keV) and O (0.525 keV), but not N (0.392 keV) which would be a weak signal and overlapped in between the C and O peaks. The presence of the Na peak in both EDX spectra came from the NaOH solution used.

### 3.2 The fluorescence turn-off sensor for ferric ions with GSH-GQDs

**Scheme 1:**
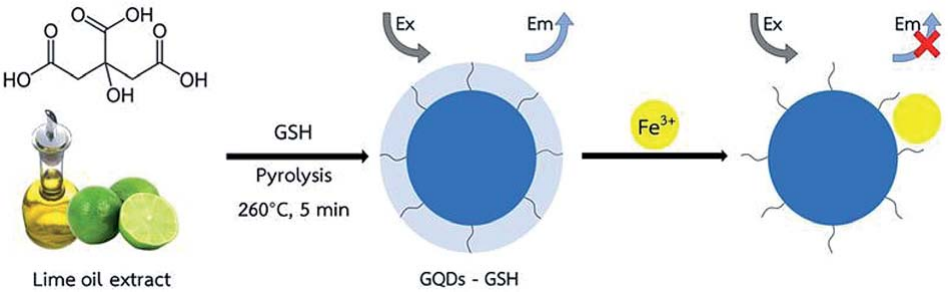
Schematic diagram for synthesizing GSH doped GQDs and their application in Fe^3+^ detection.

The overall assay strategy for the sensing of Fe^3+^ is shown in Scheme 1. GSH doped GQDs were obtained through the pyrolysis of lime oil extract in the presence of GSH at 260 °C for 5 min, in which the GSH was used as an additional sulfur and nitrogen source, while the lime oil extract was used as the natural carbon source. The absorption and emission fluorescence spectra of GSH-GQDs show peaks at 345 and 430 nm, respectively. The GSH-GQDs show excellent fluorescence stability and low toxicity under the preparation conditions.

More importantly, it was demonstrated that the GSH-GQDs were highly selective towards Fe^3+^, in which it is evident that the incorporation of S atoms into GQDs should be of crucial importance for tuning the electronic local density of the GQDs and promoting the coordination interaction between Fe^3+^ and the phenolic hydroxyl groups on the edge of S-GQDs. S (electronegativity of S: 2.58) is an electron donor, but fundamentally different from N (electronegativity of N: 3.04) because of its lower electronegativity and larger atomic radius. Therefore, the valence electrons of S in the third shell are easily lost, and the higher surface electron density of O atoms further promotes the coordination interaction.^78^ Therefore, we deduce that when Fe^3+^ ions are added into the S-GQDs solution, they can coordinate with the phenolic hydroxyl groups on the edge of S-GQDs, and the electrons in the excited state of S-GQDs will transfer to the half-filled 3d orbitals of Fe^3+^, facilitating non-radiative electron/hole recombination annihilation and leading to significant fluorescence quenching.^79,80^

### 3.3 Effect of the solution pH

**Fig. 4:**
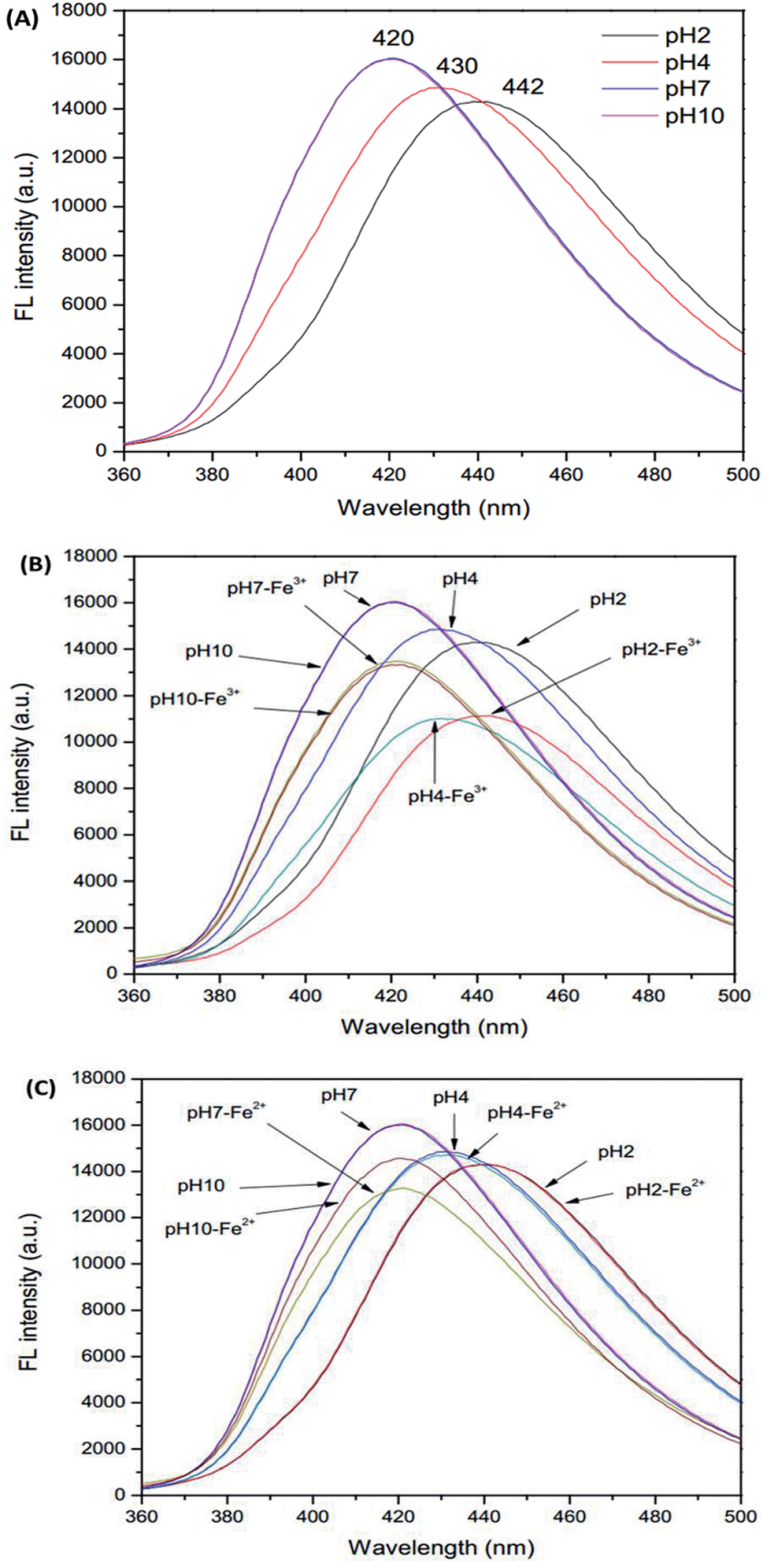
Effect of pH on (A) GSH-GQDs, (B) GSH-GQDs-Fe^3+^ and (C) GSH-GQDs-Fe^2+^.

Initially, due to the presence of pH sensitive functional groups in the GSH-GQDs, we investigated the effect of pH on their fluorescence intensity. As demonstrated in Fig. 4A–C, we found that the pH of the solution is able to influence the fluorescence signal of the sensor before and after the addition of Fe^3+^ ions. The fluorescence intensity of the sensor is plotted *versus* pH in the range of 2–10 (Fig. 4A). It was observed that the emission intensity changed the maximum wavelength and decreased sharply when the pH value was below 7. It was evident that at various pH values the fluorescence intensity was quenched by Fe^3+^, but at around pH 4 it could not be quenched by Fe^2+^. Thus, towards this sensitive pH value an iron speciation analysis could be selectively conducted in association with simple oxidation of Fe^2+^ to Fe^3+^ using H_2_O_2_ as an oxidizing agent.

In order to keep the fluorescence intensity of the GSH-GQDs as stable as possible and to ensure sensitive determination of Fe^3+^, a citrate buffer solution (pH 4, 1.0 M) was used.

### 3.4 Effect of H_2_O_2_ on speciation of Fe^2+^ and Fe^3+^

Our studies showed that Fe^3+^ ions immediately reduce the fluorescence intensity of GSH-GQDs. However, no such changes were observed when Fe^3+^ was replaced by Fe^2+^. According to the following reaction, H_2_O_2_ is capable of generating higher oxidation states of iron, which is referred to as the Fenton reaction.^81^Fe^2+^ + H_2_O_2_ → Fe^3+^ + OH^−^ + OH˙

Therefore, by means of the Fenton reaction, selective and sensitive determination of Fe^2+^ based on GSH-GQDs fluorescence quenching is feasible. The effect of H_2_O_2_ concentration was tested by evaluating the fluorescence intensity of the GSH-GQDs.

**Fig. 5:**
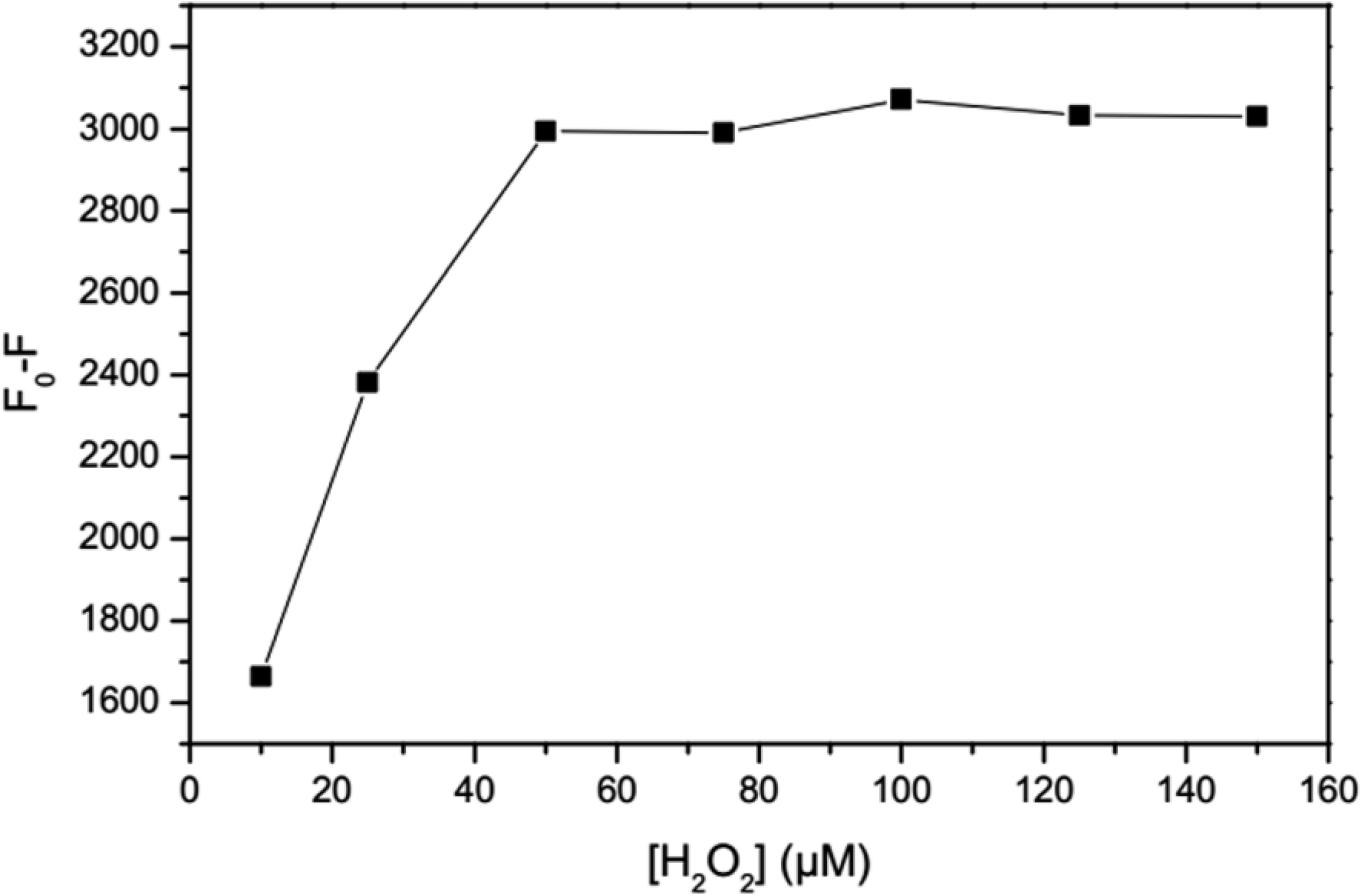
Effect of H_2_O_2_ concentration.

As shown in Fig. 5, the addition of H_2_O_2_ results in quenching of the fluorescence intensity proportional to the H_2_O_2_ concentration. The optimum concentration of hydrogen peroxide is when no obvious change in fluorescence intensity due to H_2_O_2_ is observed in the absence of Fe^2+^, while in the presence of Fe^2+^ oxidization of almost all of the Fe^2+^ ions occurs. Therefore, the effectiveness of different concentrations of H_2_O_2_ for oxidation of the highest concentration of Fe^2+^ in a linear range (100 μM) was investigated. According to the revealed results, the concentration of 50 μM was chosen for H_2_O_2_, taking the complete oxidation of Fe^2+^ ions and unnoticeable change of GSH-GQDs emission into account.

### 3.5 Effect of ionic strength and masking agent

**Fig. 6:**
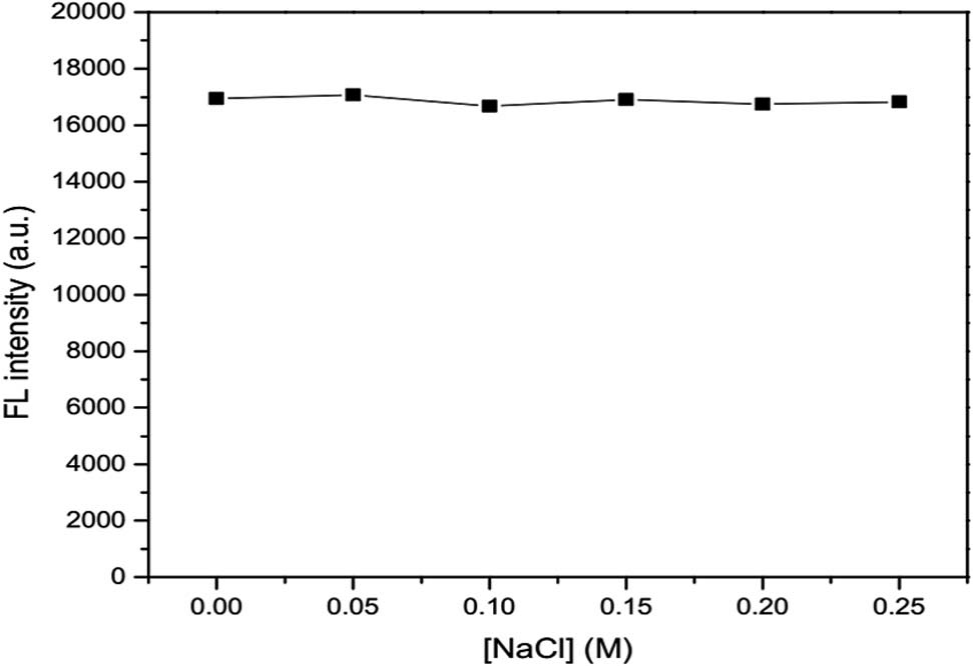
Effect of concentration of NaCl as an ionic strength.

**Fig. 7:**
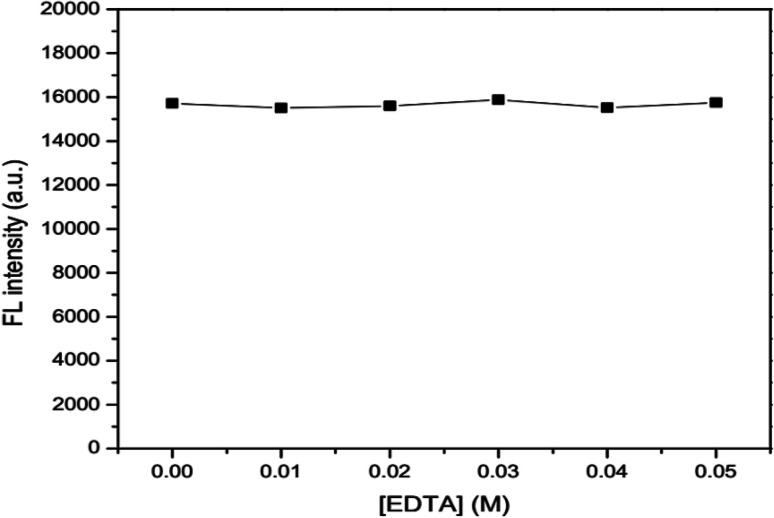
Effect of concentration of EDTA as a masking agent.

The effects of ionic strength as 0.05, 0.10, 0.15, 0.20 and 0.25 M NaCl solutions (Fig. 6) and masking agent as 0.01, 0.02, 0.03, 0.04 and 0.05 EDTA solutions (Fig. 7) tested were also investigated. It was found that both ionic strength and masking agent had no effect on the fluorescence intensity of the GSH-GQD solution. A further aim is that modification of the ionic strength of the mixture solution could be done in association with the addition of EDTA to mask any metal ion interferences in the sample.

### 3.6 Selectivity of the proposed sensor

**Fig. 8:**
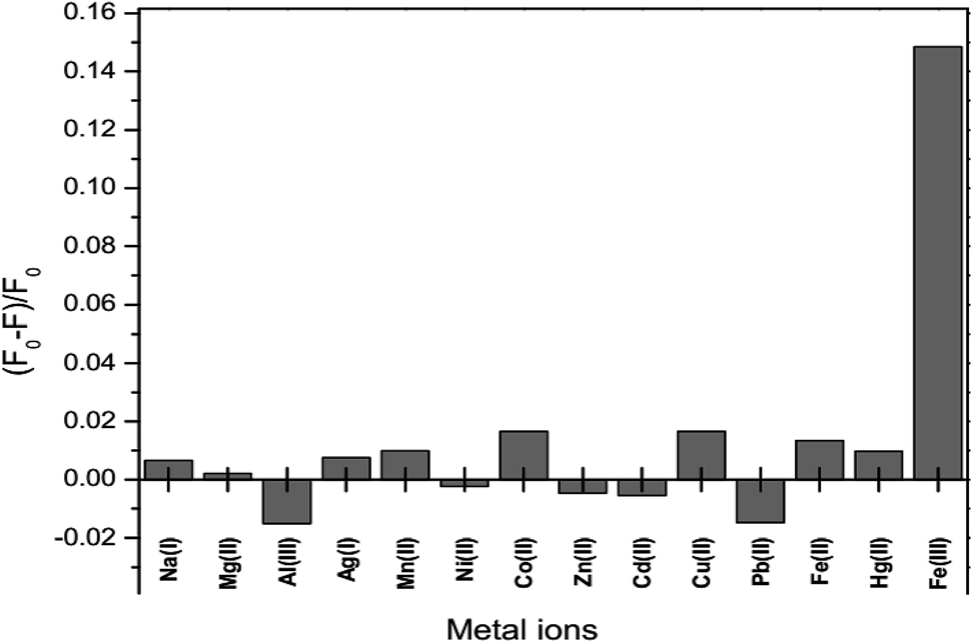
Fluorescence response of GSH-GQDs to various metal cations with a concentration of 10 μM.

The fluorescence intensity of GSH-GQDs in the presence of various metal ions, Na^+^, Ag^+^, Mg^2+^, Mn^2+^, Ni^2+^, Co^2+^, Zn^2+^, Cd^2+^, Cu^2+^, Pb^2+^ Fe^2+^, Hg^2+^, Al^3+^ and Fe^3+^, was investigated. The values of (*F*_0_ − *F*)/*F*_0_ were plotted using 0.1 mL of 10 μM of each metal. Fig. 8 shows that the addition of Fe^3+^ to the reaction mixture of the GSH-GQDs system resulted in fluorescence quenching, whereas the other remaining cations had no significant effect under the same experimental conditions.

### 3.7 Method validation

**Fig. 9:**
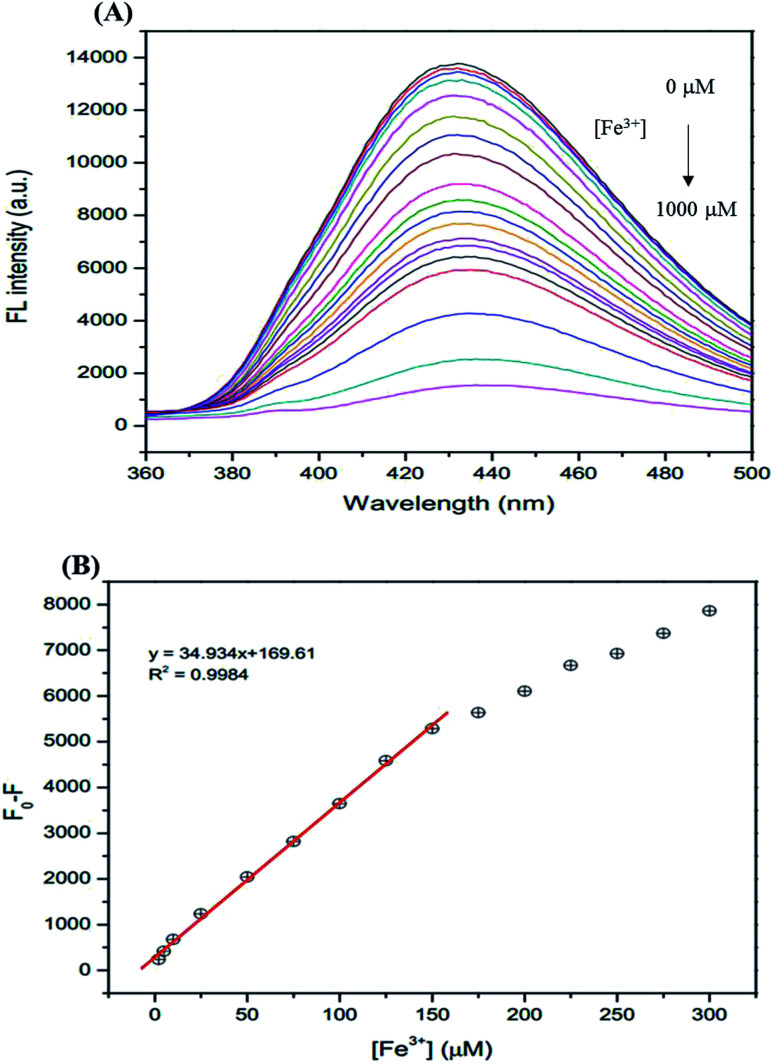
(A) The changes in the FL intensity of the GSH-GQDs at different Fe^3+^ concentrations (0–1000 μM), (B) linear relationship of *F*_0_ − *F versus* the concentration of Fe^3+^ over the range 1–150 μM.

The analytical characteristics of the proposed method were validated under optimized conditions in terms of linearity, limit of detection (LOD), limit of quantification (LOQ), and precision (expressed as the relative standard deviation, RSD, of the calibration slope obtained from both intra-day and inter-day analysis) to estimate the efficiency and feasibility of the method for use with drinking water samples. The linearity range is from 1–150 μM (*R*^2^ = 0.9984). The linear calibration graph is as follows: *y* = 34.934*x* + 169.61 (where *y* is the fluorescence intensity and *x* is the concentration of Fe^3+^) (Fig. 9). The LOD defined as 3SD/*m* (where SD is the standard deviation of a very low concentration of Fe^3+^ and *m* is the slope of the calibration graph) was 0.10 μM. The LOQ defined as 10SD/*m*, was 0.34 μM. The precision, which was evaluated in terms of repeatability (data from 3 × 3 independent standard preparation, intra-day RSD), was 2.03% and 3.17% and the reproducibility (work performed during 5 × 3 consecutive days, inter-day RSD) was 3.11% and 4.55% for Fe^2+^ and Fe^3+^, respectively, indicating acceptable repeatability of the method.

### 3.8 Real sample analysis

Iron speciation, Fe^2+^ and Fe^3+^, in some brands of bottled drinking water samples was studied. To demonstrate the applicability and reliability of the proposed method, it was successfully applied to fifteen samples of drinking water including five brands of water (brands 1–5). The amounts of Fe^2+^ and Fe^3+^ in these samples were obtained as shown in Table 1. The results showed that both Fe^2+^ and Fe^3+^ were found in all samples studied. In addition, to evaluate the matrix effect the accuracy of the method was verified by calculating the recovery in the real samples. Each sample was spiked with five concentrations (0, 25, 50, 75 and 100 μM) of a standard solution of both Fe^2+^ and Fe^3+^. Then, the relative percentage recoveries were calculated as follows:% Recovery = [(*C*_found_ − *C*_real_)/*C*_added_] × 100where, *C*_found_, *C*_real_ and *C*_added_ are the concentration of analyte after addition of a known amount of standard to the real sample, the concentration of analyte in the real sample, and the concentration of the known amount of standard that was spiked in real sample, respectively. From the results (Table 1), it was found that the recoveries of the proposed method expressed as the mean percentage (*n* = 3) were in the range of 100.09–104.09% and 98.08–102.65% for Fe^2+^ and Fe^3+^, respectively. This demonstrates that this method provides acceptable recovery for the determination of both Fe^2+^ and Fe^3+^ in real samples. Therefore, it is concluded that the matrix effect on the performance of the proposed method in these samples is negligible. The sensing property, particularly expressed as LOD, of the developed method is compared with those of selected relevant reports as shown in Table 2.^82–87^

**Table 1:** The amount and recovery of Fe^2+^ and Fe^3+^ in drinking water samples using the GQDs-GSH system (*n* = 3)

Drinking water sample	Fe^2+^			Fe^3+^		
	Added (μM)	Found (μM)	Recovery (%) ± SD	Added (μM)	Found (μM)	Recovery (%) ± SD
Brand 1	0	0.11	—	0	0.22	—
	25	25.72	102.86 ± 4.15	25	25.22	100.90 ± 1.85
	50	51.15	102.30 ± 1.08	50	49.04	98.08 ± 2.67
	75	75.95	101.27 ± 3.13	75	75.17	100.23 ± 2.54
	100	103.24	103.24 ± 4.44	100	98.55	98.55 ± 4.34
Brand 2	0	0.28	—	0	0.34	—
	25	25.51	102.05 ± 3.73	25	25.28	101.13 ± 1.89
	50	51.04	102.07 ± 2.58	50	49.99	99.99 ± 3.73
	75	78.06	104.09 ± 2.24	75	74.45	99.26 ± 5.20
	100	103.01	103.01 ± 4.11	100	98.20	98.20 ± 2.97
Brand 3	0	0.40	—	0	0.37	—
	25	25.17	100.66 ± 2.99	25	25.05	100.20 ± 2.69
	50	51.88	103.75 ± 4.34	50	49.39	98.77 ± 2.49
	75	77.69	103.58 ± 2.61	75	74.77	99.69 ± 3.15
	100	101.13	101.13 ± 3.92	100	100.06	100.06 ± 2.56
Brand 4	0	0.25	—	0	0.42	—
	25	25.02	100.09 ± 3.44	25	25.57	102.29 ± 4.22
	50	50.78	101.55 ± 4.22	50	51.33	102.65 ± 2.09
	75	76.62	102.16 ± 3.17	75	74.30	99.07 ± 5.11
	100	102.55	102.55 ± 1.97	100	99.13	99.13 ± 3.42
Brand 5	0	0.16	—	0	0.31	—
	25	25.08	100.32 ± 3.59	25	25.31	101.24 ± 3.12
	50	51.24	102.48 ± 2.99	50	50.83	101.67 ± 2.41
	75	77.28	103.04 ± 3.18	75	75.69	100.92 ± 2.38
	100	102.31	102.31 ± 3.75	100	99.51	99.51 ± 1.86

**Table 2:** Selected chemical sensors using carbon based materials for the determination of Fe^2+^ and Fe^3+^ ions

Carbon based material	Linear range	LOD	Reference
Carbon dots (CDs) conjugated with phenol groups	1–100 mM	0.17 mM	82
Gold nanoparticles (AuNPs) conjugated with glycol chitosan	0–180 μM	11.3 nM	83
Boron-doped graphene quantum dots (B-GQDs)	0.01–100 μM	0.005 μM	84
Sulfur-doped graphene quantum dots (S-GQDs)	0–0.70 μM	4.2 nM	85
Nitrogen-doped carbon dots (N-CDs)	0–1.6 μM	0.05 μM	86
N/P co-doped carbon dots	1–150 μM	0.33 μM	87
GSH-doped GQDs (lime oil extract)	1–150 μM	0.10 μM	This work

## 4. Conclusion

A highly sensitive fluorescence sensor for speciation of Fe^2+^ and Fe^3+^ ions based on the fluorescence quenching of GSH-GQDs was obtained. A novel approach for the preparation of GQDs by pyrolysis of citric acid rich-lime oil extract was demonstrated for selective determination of Fe^2+^ and Fe^3+^ at trace level under optimum conditions, mainly including ensuring the solution was pH 4.0 in the presence of H_2_O_2_. The analytical features of merit of the proposed method were well validated, and could be applied to determine both Fe^2+^ and Fe^3+^ in five brands of bottled drinking water samples, in particular having high recovery. Using the as-prepared GQDs doped with a small amount of GSH as a simple and cheap fluorescence sensor with natural lime oil extract as a starting material meets the requirements of green chemistry. In addition, using multi-functional materials such as a S- and N-doped GQD based spectroscopic sensor and/or other reactive cation or anion-doped carbon based materials with their intrinsic quantum yields could have potential in approaching green chemistry for future trends in nanoparticle applications.^88–90^

## Conflicts of interest

The authors have declared no conflict of interest.

## Supplementary Material
